# Pennsylvania State Veterinary Examining Board

**Published:** 1896-03

**Authors:** S. J. J. Harger, W. Horace Hoskins

**Affiliations:** President; Secretary


### PENNSYLVANIA STATE VETERINARY EXAMINING BOARD.

THE first stated meeting was held at the office of the Secretary, 3452 Ludlow Street, Philadelphia, on Monday, December 16, 1895. The President called the meeting to order at 11 A.M. Members present: Drs. Harger, Hoskins, and Walters. Absent: Drs. McNeil and Sallade, both of whom had forwarded their list of questions with instructions. The minutes of the initial meeting of the Board were read and approved.

The Secretary reported the correspondence relative to the cases of Drs. Kinnell and Hudson, stating that the former on investigation proved to be a graduate of the Ontario Veterinary College, legally registered in the State, and entitled to all the privileges of the several Acts. The complaint against Dr. Hudson was well substantiated, and it was quite evident that unless he ceased practising as a veterinary surgeon, it would be necessary for the Board to enter suit against him for violation of the several Acts under which the Board operated. The Secretary reported that he had investigated the grounds upon which the Prothonotary of Dauphin County had registered the said E. D. Hudson.

There being no candidates for examination, several questions came up before the Board as to the right of one legally registered in this State, removing to another State and returning to this State, as to whether his original registration would stand good or whether such a person would have to come before the Board. The matter was referred to Dr. Walters with instructions that he seek the Attorney-General's opinion upon this point.

The question of accepting the certificate of Dr. Hamilton, who had passed the State Veterinary Examining Board in Ohio, was considered, and on motion it was decided that we accept the certificate of that Board and grant him a license in this State on completion of the requirements relative to such applicants.

The By-laws were then presented as prepared by the sub-committee and taken up section by section, and were then adopted as a whole. A vote of thanks was tendered the Secretary for his labor in preparing the proposed rules.

It was moved and seconded that the meeting of the Board then adjourn to the third Monday in April, 1896, unless otherwise ordered.

S. J. J. HARGER,

President.

W. HORACE HOSKINS,

Secretary.

THE SCHUYLKILL VALLEY VETERINARY MEDICAL ASSOCIATION will hold their quarterly meeting at the Board of Health rooms, City Hall, Reading, Wednesday, March 18, 1896. Among other interesting papers and matters to come before the Association will be one on the "General Pathology of the Lymphatic System," by Dr. J. H. McCarthy; "Spavin," by Dr. M. L. Brackbill; "Mange," by Dr. U. S. G. Bieber.

THE PENNSYLVANIA STATE VETERINARY MEDICAL ASSOCIATION will have, among other interesting papers, "Remarks on the Use of Barium Chloride," by President Pearson; "The Pleasure of Being a Veterinarian," by Dr. J. Curtis Michener; "The Pathogenesis of Disease," by Dr. W. L. Rhoads. Dr. Allen will introduce the subject of "Azoturia" by reporting a second attack within five weeks, of the same horse, with recovery.

DR. JAMES A. WAUGH will enliven the programme of the coming meeting of the Pennsylvania State Association with a paper entitled "The American Veterinary Trade *versus* the Veterinary Profession."

